# Dynamic Crosslinking of LDPE by Nitroxide Radical Coupling of a Dicyclopentadiene Dicarboxylic Acid and Its Dynamic Properties

**DOI:** 10.3390/polym17111536

**Published:** 2025-05-31

**Authors:** Alojz Anžlovar, Mohor Mihelčič, Iztok Švab, David Pahovnik, Ema Žagar

**Affiliations:** 1 National Institute of Chemistry, Hajdrihova 19, SI-1000 Ljubljana, Slovenia; david.pahovnik@ki.si (D.P.); ema.zagar@ki.si (E.Ž.); 2 Faculty of Mechanical Engineering, University of Ljubljana, Aškerčeva 6, SI-1000 Ljubljana, Slovenia; mohor.mihelcic@fs.uni-lj.si; 3 ISOKON d.o.o., Industrijska Cesta 16, SI-3210 Slovenske Konjice, Slovenia; iztok.svab@isokon.si

**Keywords:** LDPE, dynamic crosslinking, reversible Diels–Alder reaction, Thiele’s acid ester, melt processing, rheology, NMR spectroscopy, FTIR spectroscopy, mechanical properties, creep

## Abstract

LDPE was crosslinked with novel dynamic or conventional crosslinking agents during melt processing. Both crosslinkers were synthesized by the esterification of Thiele’s acid or adipic acid with 4-hydroxy-TEMPO. ^1^H-NMR showed that a temperature of 170 °C and a reaction time of 24 min are required for a successful crosslinking. The concentrations of crosslinking agents were 1.45, 2.9, and 5.8 mol%. Conventionally crosslinked LDPEs show a decrease in soluble content in hot xylene with increased crosslinker concentrations, while dynamically crosslinked LDPEs show no change after thermal treatment, indicating the scission of dynamic crosslinks. The rheology of both crosslinked LDPEs at 130 °C shows that the stress release is slower than that of neat LDPE, confirming crosslinking, while at 170 °C a shift in the stress release and also a shift in the flow properties of dynamically crosslinked LDPE towards those of neat LDPE are observed, both indicating the cleavage of dynamic crosslinks. Compared to neat LDPE, the mechanical properties of both crosslinked LDPEs show an increase in Young’s modulus and tensile strength and a decrease in elongation and creep when the concentration of both crosslinkers is increased. By increasing the processing temperature to 170 °C, the crystallinity index decreases, leading to a rather small improvement in the mechanical properties.

## 1. Introduction

Dynamic covalent chemistry has been developed as a successor to supramolecular chemistry by transferring the dynamic properties of intermolecular (supramolecular) chemistry [1] to the molecular level [2,3]. This evolutionary process also influenced the field of polymer science by creating a new field of dynamic (reversible) polymer chemistry [4]. In 2011, the first paper was published dealing with the formation of a dynamically crosslinked epoxy resin utilizing the reversibility of ester bonds [5]. Since then, this field has been developing and growing rapidly along with the entire field of dynamic covalent chemistry. A large number of research groups are seeking new reversible chemical reactions or reaction systems and applying them to form various novel dynamic covalent polymers [1,6]. As reported in recent publications, not only the reprocessing, reshaping, and recycling of traditionally unprocessable thermoset polymers, but also self-healing, structural modification, processing of difficult-to-process thermosets or thermoplastic polymer materials and in-situ reinforcement of 3D–printed objects have become possible [1,7,8]. The reversible formation of covalent bonds is crucial in the production of functional materials, such as stimuli-responsive materials [9,10], porous covalent organic frameworks [11,12,13], and malleable covalent network polymers [5,14,15,16,17,18]. In addition, reversibility also introduces the adaptability of the materials that can respond to external stimuli such as the solvent, the pH of the medium, or other physical factors (light, electricity, temperature, mechanical stress, etc.) [19]. Special attention is paid to polymer networks that have reversible covalent crosslinks and are able to adapt to an externally applied stimulus such as temperature. While there are many reversible reactions that are possible crosslinking candidates in adaptable polymer networks, there are only a few that are readily and repeatedly reversible [16]. Such reactions are thermoreversible addition reactions such as the nucleophilic addition between isocyanate and imidazole, the reversible radical coupling between TEMPO and a styryl radical, and the Diels–Alder cycloaddition between furan and maleimide [16].

Such dynamically crosslinked covalent polymers or covalently adaptable networks (CANs) are divided into dissociative and associative depending on the exchange mechanism [20]. In dissociative CANs, the chemical bonds are initially broken and re-formed elsewhere, whereas in associative CANs, the original chemical bond is only broken when another chemical bond is formed at a different site. In the first type of CAN, there is an intermediate loss of crosslinks and a sudden drop in viscosity, whereas the second type of CAN does not depolymerize and retains a fixed crosslink density [20]. The first associative CANs date back to 2005 and were extended in the following years with the introduction of associative covalent chemistry in epoxy/anhydride polyester-based networks [5,15]. Polyester/polyol networks show a gradual decrease in viscosity upon heating, a characteristic feature of vitreous silica that has never been observed in organic polymer materials, so these materials are called vitrimers [21,22,23]. This idea was extended to biopolymers such as poly(lactic acid)—PLA or to renewable polymers based on epoxidized soybean oil and citric acid, as these materials are also poly(esters) [24,25,26].

Unfortunately, most of the prepared dynamic covalent polymers were synthesized in solution [6]. Such processes do not suit the synthesis and further processing of the widely used commodity polymers, which are mainly processed in the melt. So far, only a few dynamic covalent polymers have been prepared by melt processing [15,27,28,29,30,31,32]. Most of these publications deal with vitrimers based on dynamic ester bonds prepared with substantial amounts of Zn salts as a catalyst, which is not beneficial for some final properties. Conventional commodity polymers are rarely modified with dynamic covalent molecules to produce dynamically covalently crosslinked polymer materials [27,28,29,33]. Caffy et al. have studied dynamically crosslinked PE vitrimers using TEMPO-dioxaborolane moieties for dynamic crosslinking [27]. Tellers et al. have prepared dynamically crosslinked PE vitrimers using the dynamic chemistry of vinylogous urethanes and demonstrated their recyclability [31]. Ricarte et al. successfully synthesized dynamically crosslinked PE vitrimers using dynamic dioxaborolane chemistry [32]. Lopez-Villanova et al. demonstrated the formation of dynamically crosslinked PE by photochemical dimerization of anthracene [33]. In addition, reversible covalent polymers were mostly processed using methods typical of irreversibly bonded polymers, such as compression molding [34,35,36], while injection molding was rarely used [5,37,38], which is not compliant with the high-throughput manufacturing of thermoplastic polymer products. Therefore, much work still needs to be conducted to successfully introduce dynamically crosslinked or extended covalent polymers into a large-scale technology of polymer materials and into the real market.

In this work, we studied the effects of dynamic crosslinking of LDPE with a novel dicyclopentadiene crosslinker during melt processing on its chemical and physical properties compared to conventionally crosslinked LDPE using a crosslinker based on the adipic acid. Dicyclopentadiene dicarboxylic acid and adipic acid were esterified with two molecules of 4-hydroxy-TEMPO, which introduced reactive radicals into the crosslinker that reacted with LDPE chains via nitroxide radical coupling, representing an innovative approach towards crosslinking of LDPE in the melt.

## 2. Materials and Methods

### 2.1. Materials

Adipic acid (AA, Sigma-Aldrich, St. Louis, MO, USA, 99%), 4-hydroxy-TEMPO or (2,2,6,6-tetramethylpiperidin-1-yl)oxyl (HO-TEMPO, Sigma-Aldrich, St. Louis, MO, USA, 97%), *N,N*-dimethylpyridin-4-amine (DMAP, Sigma-Aldrich, St. Louis, MO, USA, 97%), 1-ethyl-3-(3-dimethylaminopropyl) carbodiimide (EDC, IRIS Biotech, Marktredwitz, Germany, 97%), dicumyl peroxide (Sigma-Aldrich, St. Louis, MO, USA, 98%), *n*-hexadecane (Sigma-Aldrich, St. Louis, MO, USA), KOH (Kemika, Zagreb, Croatia, p.a., ACS reagent), HCl (Fluka, Buchs, Switzerland, p.a., ACS reagent, fuming, >37%), MgSO_4_ (Sigma-Aldrich, St. Louis, MO, USA, anhydrous, >99.5%), isopropanol (Sigma-Aldrich, St. Louis, MO, USA, p.a.), *n*-hexane (Sigma-Aldrich, St. Louis, MO, USA, p.a., ACS reagent), ethyl acetate (Sigma-Aldrich, St. Louis, MO, USA, p.a., ACS reagent, >99.5%), dichloromethane (Sigma-Aldrich, >99.9%), Low-density polyethylene (LDPE, Dow PE-LD 352 E, Dow Europe, Leuna, Germany, Melt Index (g/10min @190 °C/2.16 kg = 2); CDCl_3_ (Sigma-Aldrich, St. Louis, MO, USA, deuteration degr. min. 99.8%); silica gel 60 (Merck, Darmstadt, Germany, 0.040–0.063 mm, for column chromatography) were used as received.

### 2.2. Synthesis of Dicyclopentadiene Dicarboxylic Acid (Thiele’s Acid or DCPD-COOH)

The synthesis of dicyclopentadiene dicarboxylic acid or Thiele’s acid (DCPD-COOH) was performed from the previously synthesized dimethyl ester of dicyclopentadiene dicarboxylic acid [39] (DCPDME) according to the procedure described in the literature [40]. DCPDME was weighed in a round bottom flask, dissolved in 10.5 mL of isopropanol, and 10 mL of 10 wt.% aqueous solution of KOH was added. The solution was mixed for 5 h at room temperature. After the reaction, the isopropanol was removed with a rotary evaporator and the resulting product was acidified to pH = 1 with concentrated HCl. The product was extracted twice with 100 mL and 50 mL ethyl acetate. The ethyl acetate solution was dried with MgSO_4_ and finally filtered to remove the desiccant. The solution was concentrated using a rotary evaporator and dried overnight in vacuum at 40 °C.

### 2.3. Synthesis of Diester of Dicyclopentadiene Dicarboxylic or Adipic Acid with (2,2,6,6-tetramethylpiperidin-1-yl)oxyl (HO-TEMPO)

Diesters (DCPD-*bis*TEMPO or AA-*bis*TEMPO) of DCPD-COOH or AA with HO-TEMPO were synthesized by esterification reaction using DMAP as catalyst and EDC as COOH group activator according to the following synthetic procedure [41,42,43]. A DCPD-COOH (0.52 g), HO-TEMPO (0.66 g), DMAP (0.65 g), and EDC (1.11 g) were weighed into a flame-dried round bottom flask (equipped with a magnet) in a dry box and sealed with rubber septa. After removing the flask from the dry box, 10 mL of dichloromethane was added with a syringe and mixing was started. The reaction was performed for 24 h at room temperature. This solution was extracted three times with 60 mL of brine, dried with MgSO_4,_ and finally filtered to remove the desiccant. The resulting solution was concentrated on a rotary evaporator and placed on a silica gel column. Column chromatography was performed using the hexane/ethyl acetate (3/1) solvent system to obtain three fractions corresponding to the three isomers of the DCPD esters. The solvent was removed with a rotary evaporator, and the final products (isomers) were dried overnight in vacuum at 40 °C. The same procedure was used for the synthesis of the diester of adipic acid with HO-TEMPO l (AA-*bis*TEMPO).

### 2.4. Melt Processing of LDPE Crosslinked with DCPD-bisTEMPO or AA-bisTEMPO Testing Samples

For melt processing, LDPE pellets were first weighed and then calculated amounts of DCPD-*bis*TEMPO or AA-*bis*TEMPO and DCP were added. The concentrations of the crosslinker were 1.45, 2.9, and 5.8 mol%. The mole percentage of DCP was half of the crosslinker concentration. The prepared granulates were extruded at 170 °C for 24 min at 100 rpm with a Haake MiniLab extruder (Thermo Fischer Scientific, Karlsruhe, Germany). The mixture was fed into the extruder in two 3.5 g portions. The extruded melt was ejected from the extruder at 250 rpm, collected in a heated container (170 °C), and fed into a Haake Mini Jet molding machine (Thermo Fischer Scientific, Karlsruhe, Germany) to prepare the test specimens by injection into a suitable mold heated to 70 °C at a pressure of 750 bar and a time of 10 s, as well as the post pressure of 250 bar and time of 10 s. Dog bone specimens for tensile tests, spherical platelets for rheological measurements, and standard platelets for DMA measurements were prepared according to the described procedure.

### 2.5. Model Reactions of the Crosslinking

We also performed the simulation of LDPE crosslinking in the DSC calorimeter. *n*-Hexadecane was used as a model compound for LDPE, DCPD-*bis*TEMPO as a crosslinker, and DCP as an initiator. Typically, 20 mg *n*-hexadecane, 3 mg DCPD-*bis*TEMPO, and 0.95 mg DCP were weighed into DSC pans, mixed, and heated under conditions similar to the melt processing of the samples. The DSC experiments were performed for 12, 24, and 36 min at 150 and 170 °C. After the experiments, the DSC pans were opened and the resulting resinous materials were dissolved in CDCl_3_ and the solutions were used for ^1^H NMR analyses.

### 2.6. Characterization

The differential scanning calorimetry (DSC) curves were recorded on a DSC-1 calorimeter (Mettler Toledo, Greifensee, Switzerland) in the temperature range from 25 °C to 180 °C at a heating rate of 10 K/min in an N_2_ atmosphere to evaluate the melting temperatures and enthalpies. The crystallinity indices were calculated according to the literature [27].

The chemical structure of the synthesized DCPD-*bis*TEMPO and AA-*bis*TEMPO crosslinkers was studied by Fourier-transform infrared spectroscopy (FTIR) on a Spectrum One FTIR spectrometer (Perkin-Elmer, Waltham, Massachusetts, USA) in transmission mode in the spectral range between 400 and 4000 cm^−1^ and with a spectral resolution of 4 cm^−1^. The samples were prepared using the KBr pellet technique.

Proton nuclear magnetic resonance (^1^H NMR) spectra of *n*-hexadecane as a model compound for PE chains crosslinked with DCPD-TEMPO were recorded using a Bruker 600-MHz spectrometer (Billerica, Massachusetts, USA) under the following quantitative conditions: pulse 90°, pre-scan delay time 6.5 μs, acquisition time 2.75 s or 2.0 s. The samples were dissolved in CDCl_3_.

The rheological behavior of polymer melts of differently crosslinked LDPEs was studied at temperatures of 130 °C and 170 °C. Injection-molded, disk-shaped samples were used for testing. All measurements were performed on an Anton Paar MCR 302 rheometer (Anton Paar, Graz, Austria) equipped with a parallel plate geometry of 25 mm diameter and 1 mm distance between the plates. Stress relaxation measurements were carried out at 130 °C and 170 °C by applying a constant shear stress of 1 MPa for 1000 s. Standard rotational flow tests were performed by changing the shear stress from 0.1 MPa to 10–30 MPa, depending on the measurement temperature.

The mechanical properties were measured according to the ISO 527 standard on the Shimadzu AGS-GX (Shimadzu, Kyoto, Japan) plus a dynamometer equipped with the ME46-NG axial extensometer using pretension of 10 N at an initial distance of 58 mm and a test speed of 1 mm/min (from 0 to 0.25% extension) and 50 mm/min (from extension 0.25% on).

Creep measurements of crosslinked LDPE samples were performed with the MCR 702 modular rheometer (Anton Paar, Graz, Austria) coupled with solid rectangular fixtures (SRF sensor) and a CTD180 temperature chamber. For the characterization, injection-molded rectangular-shaped samples with the dimensions: 35 mm length, 10 mm width, and 1 mm thickness were prepared for the measurements and a measuring length was 20 mm. The measurements were carried out at a temperature of 80 °C in three repetitions for each polymer composite. The samples were heated from room temperature to 80 °C and held at 80 °C for 1 h prior to measurement. The creep test was then performed at 80 °C for 1 h with a force of 15 N applied to the sample.

## 3. Results and Discussion

**Figure 1:**
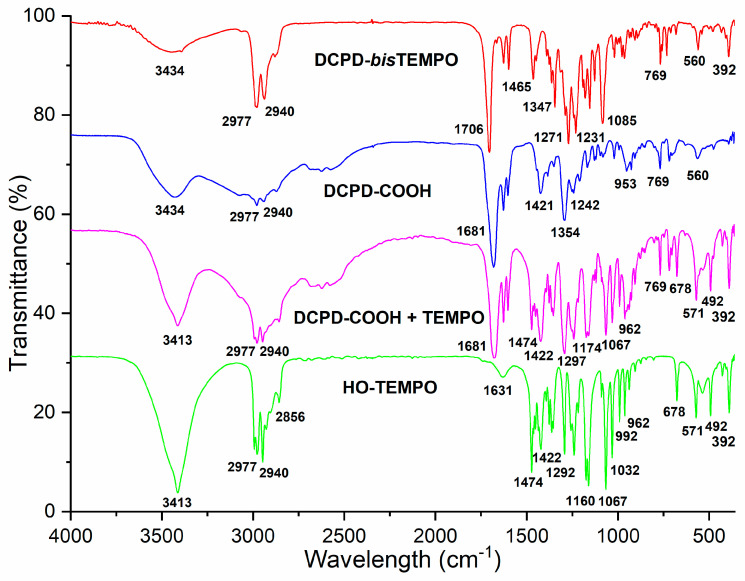
FTIR spectra of HO-TEMPO, DCPD-COOH, a mixture of DCPD-COOH and HO-TEMPO and DCPD-*bis*TEMPO ester.

The dynamic crosslinking agent used to crosslink LDPE was synthesized in two steps. The first step includes the synthesis of DCPD-COOH according to the procedure described in the literature [39,40]. The diesters of DCPD-COOH or AA with HO-TEMPO were synthesized by a Steglich esterification reaction using DMAP as a catalyst and EDC as an activator of the carboxyl group according to the procedure described in the literature [41,42,43]. The complete synthetic process is shown in Figure S1A. This synthesis was repeated several times and the yields varied between 35% and 50%. Rather low yields can be explained by side reactions of Steglich esterification [44]. The FTIR spectrum of the product DCPD-*bis*TEMPO is shown in comparison to the mixture of DCPD-COOH and HO-TEMPO and pure DCPD-COOH and pure HO-TEMPO (Figure 1). During the reaction, an ester bond is formed as indicated by the C=O absorption band at 1706 cm^−1^, in OK! contrast to the carboxylic carbonyl absorption band at 1681 cm^−1^ in the case of DCPD-COOH or the DCPD-COOH + TEMPO mixture (Figure 1). With the aim to compare the dynamic crosslinking of the LDPE with a conventional non-dynamic crosslinking, a crosslinking agent was also synthesized from AA and HO-TEMPO using the same catalyst and activator (AA-*bis*TEMPO, Figures S1B and S2).

**Figure 2:**
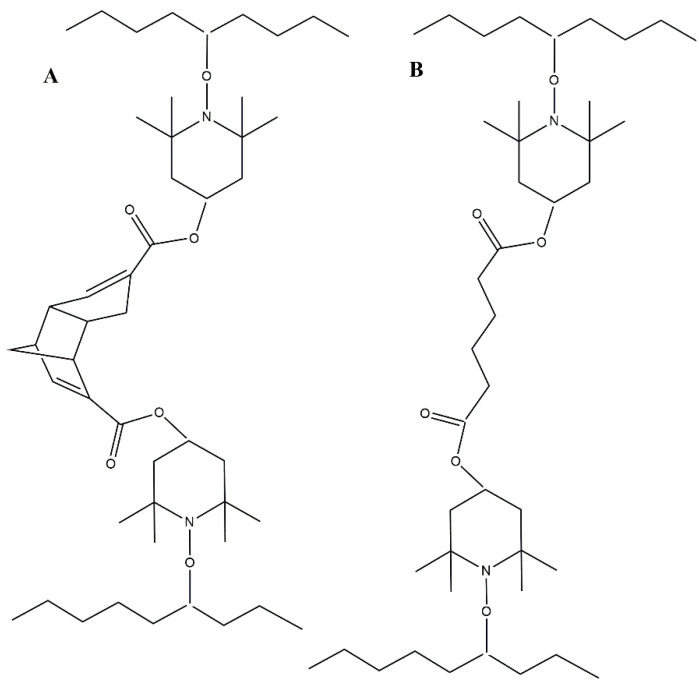
Schematic representation of LDPE crosslinking: (**A**) dynamic with DCPD-*bis*TEMPO and (**B**) conventional with AA-*bis*TEMPO.

Both crosslinkers and dicumyl peroxide (DCP) were added to the LDPE pellets before melt processing. At temperatures above 150 °C, DCP decomposes into two radicals, which are transferred to the LDPE chain and further react with the radicals from TEMPO to crosslink the LDPE polymer chains (Figure 2A,B). The dicyclopentadiene (DCPD) part of the crosslinks in Figure 2A has dynamic properties, meaning that dicyclopentadiene is reversibly cleaved into two cyclopentadiene monomers [40].

**Figure 3:**
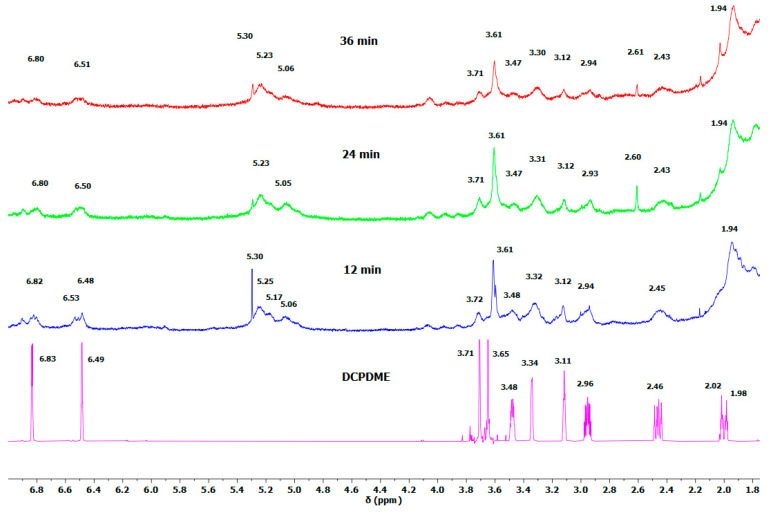
Enlarged ^1^H NMR spectra of DCPDME and the mixture of *n*-hexadecane, DCP, and DCPD-TEMPO after heating at 150 °C for 12, 24, and 36 min (Samples were dissolved for 48 h).

**Figure 4:**
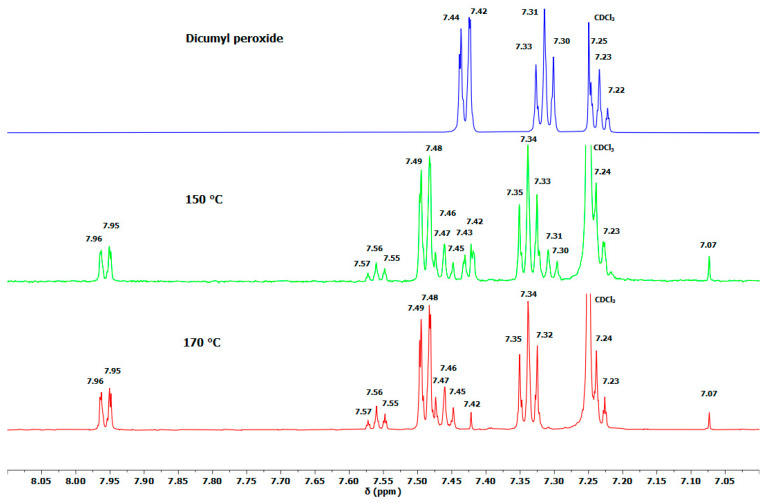
Enlarged ^1^H NMR spectra of DCP at RT and of the mixture of DCP and DCPD-TEMPO after heating at 150 and 170 °C for 12 min.

The chemistry of LDPE crosslinking with DCPD-*bis*TEMPO or AA-*bis*TEMPO using dicumyl peroxide as an initiator was studied by ^1^H NMR spectroscopy. We used *n*-hexadecane as a model compound for LDPE. These three compounds were weighed into DSC pans in the appropriate ratio, mixed, and heated under conditions similar to the melt processing of the samples. The DSC experiments were performed for 12, 24, and 36 min at 150 and 170 °C. The resulting crosslinked *n*-hexadecane was dissolved in CDCl_3_ and ^1^H NMR spectra were recorded. Figure 3 shows the ^1^H NMR spectra of DCPDME and the spectra of a mixture of *n*-hexadecane, dicumyl peroxide, and DCPD-*bis*TEMPO after 12, 24, and 36 min of heating. In the range of 1.8–7.0 ppm, DCPD signals can be seen that match well with the signals of pure DCPDME (Figure 3) [40]. Furthermore, additional signals at 5.06, 5.17, and 5.23 ppm are observed in these spectra (Figure 3), which correspond to methine protons on the *n*-hexadecane chain formed during the crosslinking reaction (Figure 2A,B). This confirms that the crosslinking of the alkyl chains takes place. Their intensities relative to the signal of the LDPE chain at 1.25 ppm are 0.0027 (12 min), 0.0029 (24 min), and 0.0043 (36 min). Their rather low intensities are ascribed to the lack of mixing and high viscosity of the system as well as to the stability of DCP at 150 °C. Figure S3 shows intense signals (7.2–7.6 ppm) from the protons of the aromatic ring of dicumyl peroxide (DCP). It can be seen that after 12 min, about half of the DCP had not yet reacted, but after 24 min all DCP was consumed, indicating that the reaction time of 12 min is too short (Figure S3). The enlarged region of ^1^H NMR (0.5–2.0 ppm) in Figure S4 shows the signal of the methyl group of DCP at 1.55 ppm. After 12 min reaction time, besides the signal at 1.55 due to neat DCP, a new signal at 1.59 ppm appeared, which originates from the reacted DCP and matches the aromatic proton signals in the range 7.2–7.8 ppm. After 24 and 36 min, only a signal at 1.59 ppm can be seen, indicating that the entire DCP has reacted (Figure S4). In addition, we performed a similar DSC experiment for 12 min at 150 and 170 °C, and the ^1^H NMR spectra of the products were recorded (Figure 4). The signals of unreacted DCP practically disappeared at 170 °C after 12 min of heating, which leads us to the conclusion that the suitable processing temperature is 170 °C and we additionally opted for a processing time of 24 min to achieve successful crosslinking of LDPE and a more uniform distribution of the crosslinkers in the LDPE matrix during melt processing.

**Figure 5:**
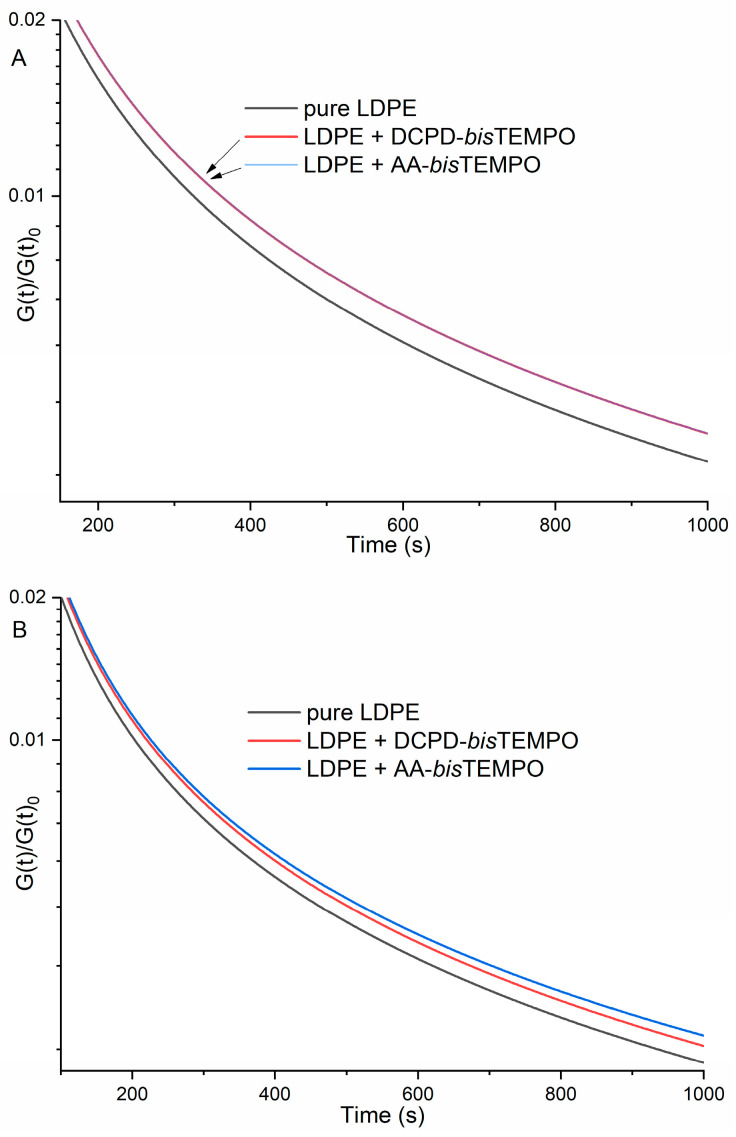
Stress relaxation profiles of neat LDPE, LDPE crosslinked with DCPD-*bis*TEMPO and LDPE crosslinked with AA-*bis*TEMPO at: (**A**) 130 °C and (**B**) 170 °C (crosslinker concentration in both cases: 5.8 mol%).

**Figure 6:**
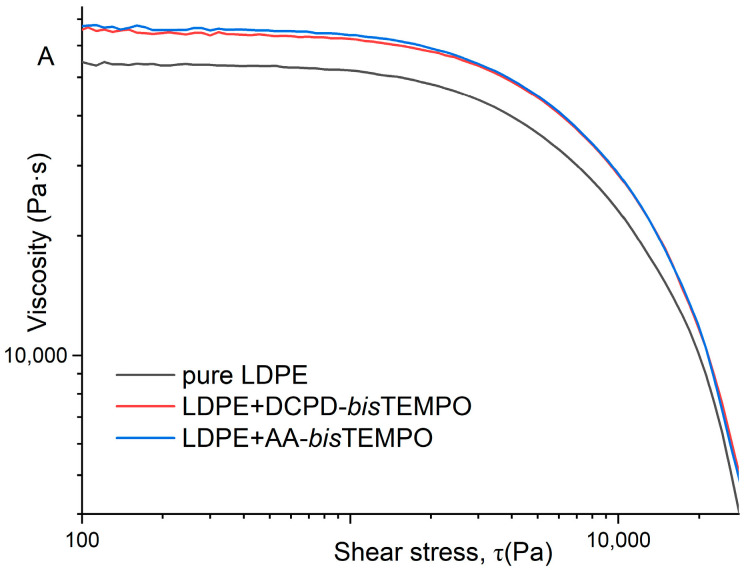

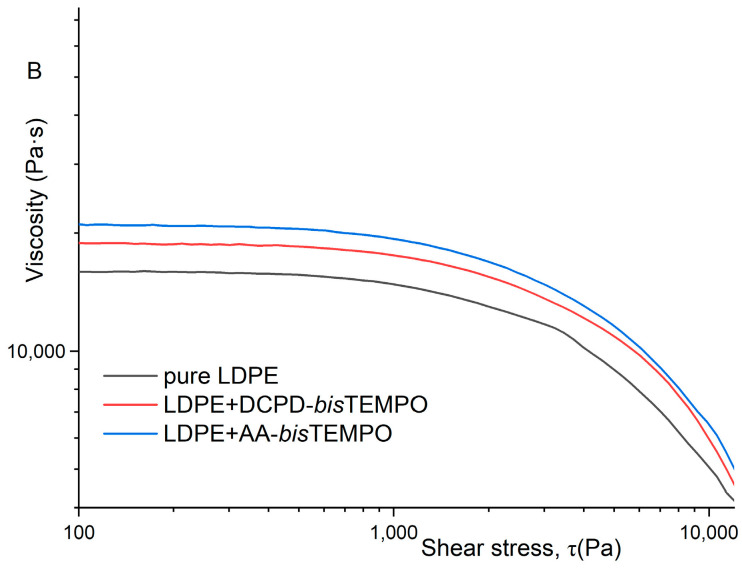
Flow properties as a function of shear stress for neat LDPE, LDPE crosslinked with DCPD-*bis*TEMPO and LDPE crosslinked with AA-*bis*TEMPO at: (**A**) 130 °C and (**B**) 170 °C (crosslinker concentration in both cases: 5.8 mol%).

The rheological properties of LDPE and LDPE crosslinked with 5.8 mol% DCPD-*bis*TEMPO or AA-*bis*TEMPO were also studied. Figure 5A shows the stress relaxation profiles for neat LDPE and both crosslinked LDPEs at 130 °C. Both crosslinked samples release stress more slowly than neat LDPE, indicating a certain degree of crosslinking and the differences between the two types of crosslinking are so small that the curves overlap. The stress relaxation profiles of neat LDPE and LDPE crosslinked with DCPD-*bis*TEMPO or AA-*bis*TEMPO at 170 °C are shown in Figure 5B [40]. The stress relaxation of LDPE crosslinked with DCPD-*bis*TEMPO is faster than that of LDPE crosslinked with AA-*bis*TEMPO, indicating that the dynamic crosslinking is interrupted in the former sample as expected (Figure 5B). The stress release of the neat LDPE was the fastest, as expected, since it is not crosslinked (Figure 5B). This behavior is similar to that reported in the literature, although the intensity of the changes is lower [27]. The viscosity as a function of shear stress also shows only slight differences for the two crosslinked LDPEs at 130 °C, while the values for neat LDPE are much lower (Figure 6A). At 170 °C, the viscosity values of LDPE crosslinked with DCPD-*bis*TEMPO are lower than those of LDPE crosslinked with AA-*bis*TEMPO, but still higher than those of neat LDPE (Figure 6B), which can be attributed to the decomposition of the DCPD moieties at 170 °C and the loss of crosslinks in this sample.

The mechanical properties of the LDPE and dynamically crosslinked LDPE at 170 °C are summarized in Table 1, while the results for the same samples processed at 150 °C are summarized in Table S1. We assumed that the crosslinking takes place predominantly in the amorphous domains of LDPE [32]. The moduli of the samples processed at 150 and 170 °C are shown in Figure S5A and in Figure S6A, respectively. A comparison of the elastic moduli in Table 1 and Figure S6A shows that the modulus increases with the concentration of AA-*bis*TEMPO and DCPD-*bis*TEMPO crosslinker. The elastic moduli of the samples processed at 150 °C show rather constant values for the LDPE crosslinked with AA-*bis*TEMPO and a slight decrease for the LDPE crosslinked with DCPD-*bis*TEMPO (Table S1, Figure S5A). The explanation for this is that crosslinking is more effective at 170 °C than at 150 °C. The maximum observed enhancement of the Young’s modulus of the samples processed at 170 °C is 5.7% (Figure S6A). The tensile strengths of DCPD-*bis*TEMPO crosslinked LDPE and those of AA-*bis*TEMPO crosslinked LDPE processed at 170 °C show an initial increase of 7.9% and 8.3%, respectively, and a maximum increase of 8.9 or 10.5%, respectively, compared to the value of LDPE (Table 1, Figure S6B). This can be explained by the fact that the crosslinkers are not uniformly distributed in the LDPE matrix at higher concentrations due to their incompatibility with LDPE which causes microphase separation, leading to the formation of a network composed of crosslinked aggregates and fractals in the LDPE material, resulting in disproportionate increase in tensile strength with the crosslinker concentration (Table 1, Figure S6B) [45]. The tensile strengths of samples processed at 150 °C showed only insignificant changes with the concentration of both crosslinkers (Table S1, Figure S5B) due to low crosslinking efficiency. The elongation at break of the samples processed at 170 °C decreases with increasing crosslinker concentration (Table 1), while the samples processed at 150 °C show no significant trend (Table S1). All these results confirm that the crosslinking of LDPE at 170 °C is more intense than at 150 °C and this is consistent with the results of ^1^H NMR spectroscopy (Figure 4).

Table 2 shows the creep behavior of dynamically and non-dynamically crosslinked LDPE compared to neat LDPE. The creep slightly decreases with increasing crosslinker concentration. The maximum decrease for LDPE crosslinked with DCPD-*bis*TEMPO and AA-*bis*TEMPO is 10.7% and 9.5%, respectively, confirming successful crosslinking of LDPE during melt processing. These changes in creep behavior are similar to those published for HDPE crosslinked with dioxaborolane, but the magnitude of the changes is much smaller in our case [27]. Additionally, Table 2 shows the melting enthalpies and crystallinity fractions of the LDPE samples processed at 150 °C and 170 °C. The crystallinity indices of all samples are between 35 and 40%, which is in accordance with literature data [46]. The differences are relatively small, but show a trend towards decreasing melting enthalpy and thus decreasing degree of crystallinity (Table 2, Figure S7), most probably as the crosslinking agent incorporated into the LDPE structure acts as an imperfection causing the decrease in crystallinity index [32]. The crystallinity indices of the samples processed at 170 °C are app. 10% lower than those of the samples processed at 150 °C (Table 2, Figure S7). This is consistent with published results for LDPE, where it was observed that the crystallinity index decreases at melt temperatures above 150 °C [47,48,49]. From this, we conclude that crosslinking with both types of crosslinkers only slightly reduces the crystallinity degree of LDPE because there are many amorphous domains where crosslinking can occur, but the processing conditions significantly influence its crystallinity index (Figure S7).

In general, the results of the rheological study, tensile, and creep tests confirm the crosslinking of LDPE at 170 °C, while the degree of crosslinking at 150 °C is much lower, as the DCP initiator is relatively stable at 150 °C and effectively decomposes into radicals at 170 °C. Rheological behavior also confirms the dynamic crosslinking of LDPE by DCPD-*bis*TEMPO at 170 °C. A comparison with the reported results on HDPE shows similar changes in rheological and mechanical properties with increasing crosslinker concentration, but in our case, the impact is much smaller, although the maximum crosslinker concentration is higher, which is most probably a consequence of the lower degree of crosslinking [27]. The results of the mechanical properties show that the LDPE matrix becomes saturated at concentrations of crosslinker higher than 2.9 mol.% due to its incompatibility with LDPE, and a further increase in concentration has a rather small effect on the crosslinking degree and on mechanical properties. Increasing the processing temperature to 170 °C enables the successful crosslinking of LDPE, but also significantly reduces the crystallinity index (Figure S7). Thus, the crosslinking effect compensates for the decrease in crystallinity, as the mechanical properties show positive trends (Figure S6A,B), which is an additional explanation for the rather small increase in mechanical properties (Table 1) and the decrease in creep behavior (Table 2). Therefore, when studying the properties of crosslinked, semi-crystalline polymers such as PE, not only the degree of crosslinking is important, but also the crystallinity index.

## 4. Conclusions

Novel dynamic and conventional crosslinkers were successfully synthesized by esterification of DCPD-COOH or adipic acid with HO-TEMPO, as demonstrated by FTIR. These two types of crosslinkers were used for LDPE crosslinking via nitroxide radical coupling reaction during melt processing. The chemistry of LDPE crosslinking was studied by ^1^H NMR spectroscopy while crosslinking reactions were performed in the DSC calorimeter using *n*-hexadecane as a model compound for LDPE and optimal reaction conditions were determined (170 °C and 24 min reaction time). ^1^H NMR spectroscopy confirmed the crosslinking of the alkyl chains of LDPE.

The crosslinking of LDPE as a function of crosslinker concentration was performed during melt processing at 170 °C for 24 min at 100 rpm, and then the test samples were injection-molded at 150 or at 170 °C and a pressure of 750 bar.

A rheological study shows that the stress release of crosslinked LDPEs at 130 °C is independent of the type of crosslinker and occurs more slowly than with non-crosslinked LDPE, which confirms a certain degree of crosslinking. The viscosity as a function of shear stress for the two crosslinked LDPEs at 130 °C also shows only slight differences, while the values for neat LDPE are lower. At 170 °C, a shift of the stress release curve of the dynamically crosslinked LDPE towards the curve of the neat LDPE can be observed, while its viscosity as a function of shear stress also decreases towards the viscosity of the neat LDPE, both again indicating the splitting of the dynamic crosslinks in this sample.

The mechanical properties of the crosslinked LDPEs show an increase in Young’s modulus and a decrease in elongation at break compared to the non-crosslinked LDPE when the concentration of the two crosslinkers increases. In addition, tensile strength increases and creep decreases in the same order, confirming the successful crosslinking of LDPE. These changes are not significant, but the concentrations of the added crosslinkers are also rather low. The changes in crystallinity index with increasing crosslinker concentration are also rather small, which is expected since the crystallinity degree of LDPE is between 35% and 40%, which means that there are many amorphous domains where the crosslinking reaction can take place without affecting the crystallinity index. However, the crystallinity index decreases considerably when the processing temperature increases from 150 to 170 °C, which also explains the rather small and insignificant increase in the Young’s modulus and tensile strength with the increase in the degree of crosslinking, as the increase in mechanical properties due to crosslinking compensates for their decrease due to the lower degree of crystallinity. This implies that when studying semi-crystalline polymers, the changes in the degree of crystallinity and not just the degree of crosslinking should always be considered.

## Figures and Tables

**Table 1:** Mechanical properties of dynamically and non-dynamically crosslinked LDPEs processed at 170 °C as a function of the crosslinker concentration (DCPD-*bis*TEMPO or AA-*bis*TEMPO).

Sample Designation	Conc. of Crosslinker	Young’s Modulus	Tensile Strength	Elongation at Break
	mol.%	GPa	MPa	%
LDPE	0	0.210 ± 0.03	15.9 ± 1.0	56 ± 8
DCPD	1.45	0.209 ± 0.02	17.2 ± 0.7	50 ± 5
DCPD	2.9	0.216 ± 0.03	17.3 ± 0.9	50 ± 7
DCPD	5.8	0.216 ± 0.02	17.4 ± 0.6	44 ± 8
AA	1.45	0.210 ± 0.03	17.3 ± 0.5	48 ± 8
AA	2.9	0.222 ± 0.02	17.6 ± 0.6	45 ± 6
AA	5.8	0.220 ± 0.03	17.6 ± 0.6	42 ± 6

**Table 2:** Creep and thermal properties of dynamically and non-dynamically crosslinked LDPE samples as a function of the crosslinker concentration (Processing temperature = 150 and 170 °C).

Sample Designation	Conc. of Crosslinker	Creep 170 °C	Melting Enthalpy 170 °C	Melting Enthalpy 150 °C	Crystal. Index 170 °C	Crystal. Index 150 °C
	Mol. %	%	J/g	J/g	%	%
LDPE	0	3.56 ± 0.2	105.0	114.3	36.6	39.7
DCPD	1.45	3.46 ± 0.3	104.3	114.1	36.2	39.6
DCPD	2.9	3.24 ± 0.2	102.8	112.7	35.6	39.5
DCPD	5.8	3.18 ± 0.2	102.1	111.2	35.4	38.5
AA	1.45	3.46 ± 0.3	104.1	114.1	36.1	39.6
AA	2.9	3.42 ± 0.4	103.8	112.8	36.0	39.1
AA	5.8	3.22 ± 0.2	103.4	113.7	35.8	39.4

## Data Availability

Data is contained within the article or Supplementary Materials.

## Supplementary Materials

The following supporting information can be downloaded at: https://www.mdpi.com/article/10.3390/polym17111536/s1, Figure S1. Synthetic procedures for preparation of DCPD-*bis*TEMPO (A) and ADK-*bis*TEMPO (B) crosslinking agents; Figure S2. FTIR spectra of adipic acid (AA), HO-TEMPO, a mixture of AA and HO-TEMPO and AA esterified with HO-TEMPO (AA-*bis*TEMPO ester); Figure S3. ^1^H NMR spectrum of the DCP at RT and ^1^H NMR spectra of the mixture of DCP and DCPD-*bis*TEMPO after 12, 24, and 36 min of heating at 150 °C (Range from 6.0 to 8.0 ppm); Figure S4. ^1^H NMR spectrum of the DCP at RT and ^1^H NMR spectra of the mixture of DCP and DCPD-*bis*TEMPO after 12, 24, and 36 min of heating at 150 °C (Range from 0.5 to 2.0 ppm); Table S1. Mechanical properties of dynamically and conventionally crosslinked LDPE samples processed at 150 °C as a function of the crosslinker concentration; Figure S5. Young’s modulus (A) and tensile strength (B) of LDPEs processed at 150 °C as a function of the crosslinker concentration (DCPD-*bis*TEMPO or AA-*bis*TEMPO); Figure S6. Young’s modulus (A) and tensile strength (B) of LDPEs processed at 170 °C as a function of the crosslinker concentration (DCPD-*bis*TEMPO or AA-*bis*TEMPO); Figure S7. Crystallinity index as a function of the crosslinker concentration and the processing temperature.
