# Supplementary material for: Dynamic Crosslinking of LDPE by Nitroxide Radical Coupling of a Dicyclopentadiene Dicarboxylic Acid and Its Dynamic Properties
Source: Polymers (Basel). 2025 May 31;17(11):1536. doi: 10.3390/polym17111536 (PMC12157318; doi:10.3390/polym17111536)
Supplement: Supplementary file 1 [file polymers-17-01536-s001.zip › polymers-3644012-supplementary.pdf]

## Supplementary Materials

# Dynamic Crosslinking of LDPE by Nitroxide Radical Coupling of a Dicyclopentadiene Dicarboxylic Acid and Its Dynamic Properties


**Alojz Anžlovar [1,*], Mohor Mihelčič [2], Iztok Švab [3], David Pahovnik [1] and Ema Žagar [1]**

[1] National Institute of Chemistry, Hajdrihova 19, SI-1000 Ljubljana, Slovenia; david.pahovnik@ki.si (D.P.); ema.zagar@ki.si (E.Ž.)

[2] Faculty of Mechanical Engineering, University of Ljubljana, Aškerčeva 6, SI-1000 Ljubljana, Slovenia; mohor.mihelcic@fs.uni-lj.si (M.M.)

[3] ISOKON d.o.o., Industrijska Cesta 16, SI-3210 Slovenske Konjice, Slovenia; iztok.svab@isokon.si (I.Š.)

* Correspondence: alojz.anzlovar@ki.si (A.A.); Tel.: +386-1-4760-204


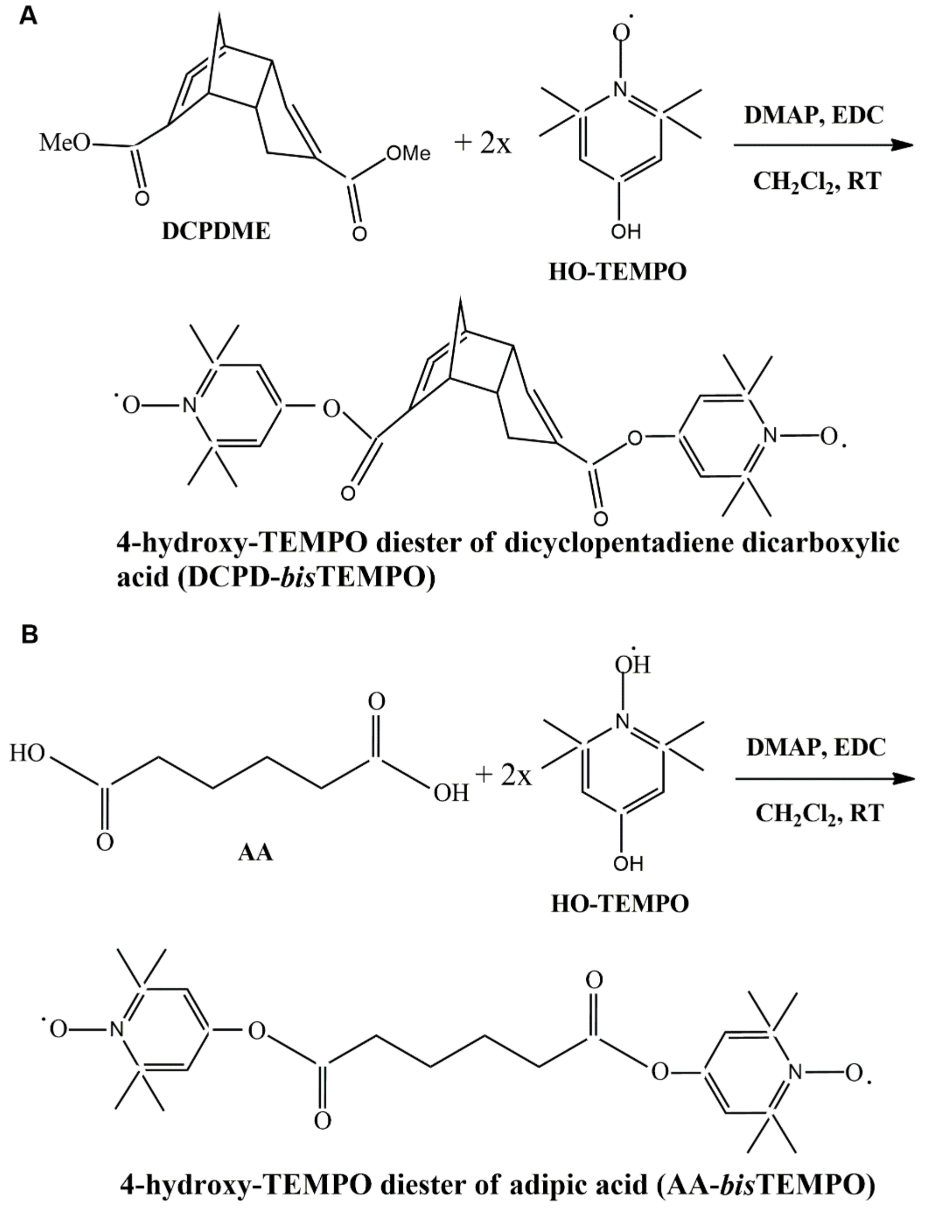

**Figure S1.** Synthetic procedures for the preparation of DCPD-*bis*TEMPO (**A**) and AA-*bis*TEMPO (**B**) cross-linking agents.

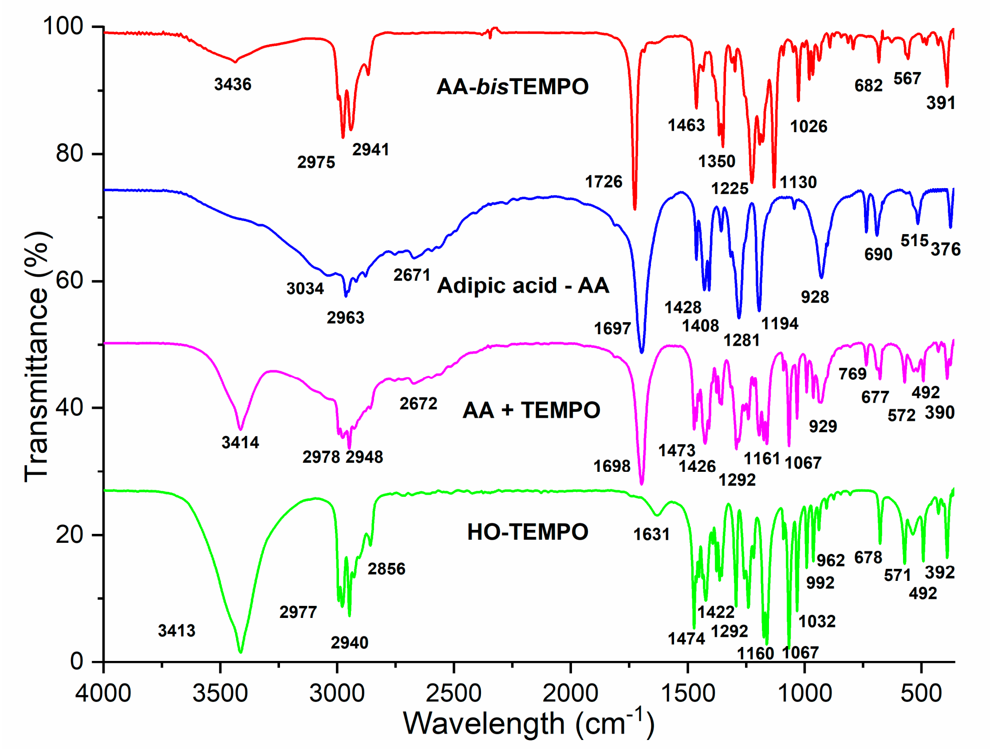


**Figure S2.** FTIR spectra of adipic acid (AA), HO-TEMPO, mixture of AA and HO-TEMPO and AA esterified with HO-TEMPO (AA-*bis*TEMPO ester).

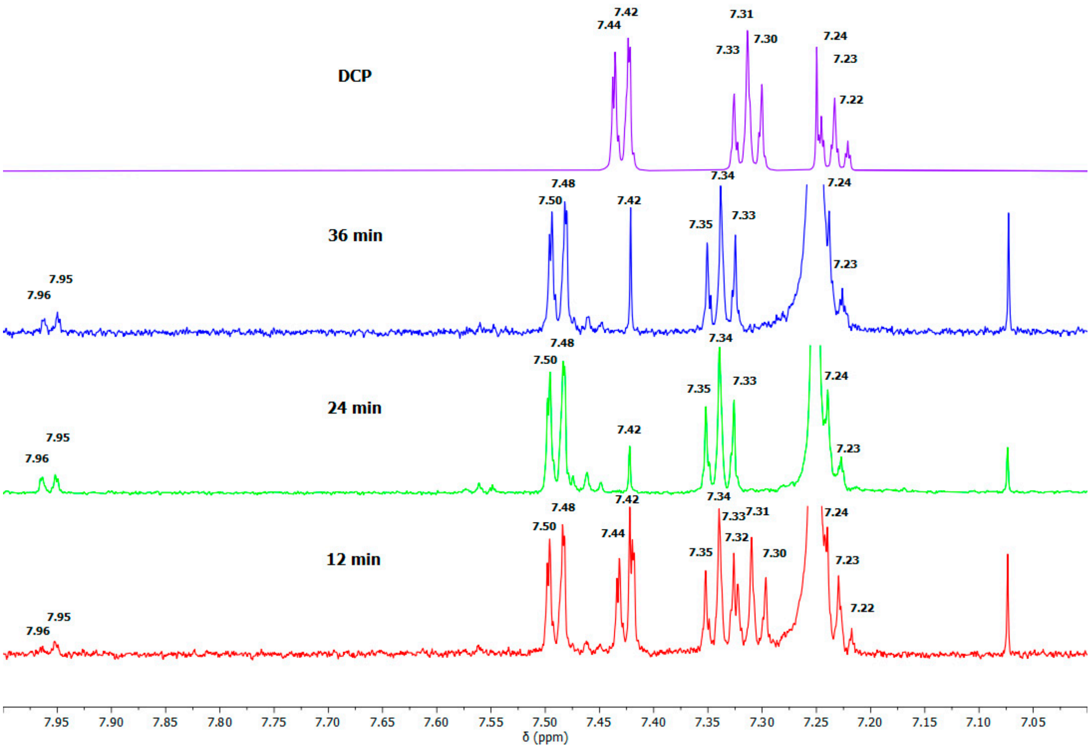

**Figure S3.** $^1$H NMR spectrum of the DCP at RT and $^1$H NMR spectra of the mixture of DCP and DCPD-*bis*TEMPO after 12, 24, and 36 min of heating at 150 °C (Range from 6.0 to 8.0 ppm).

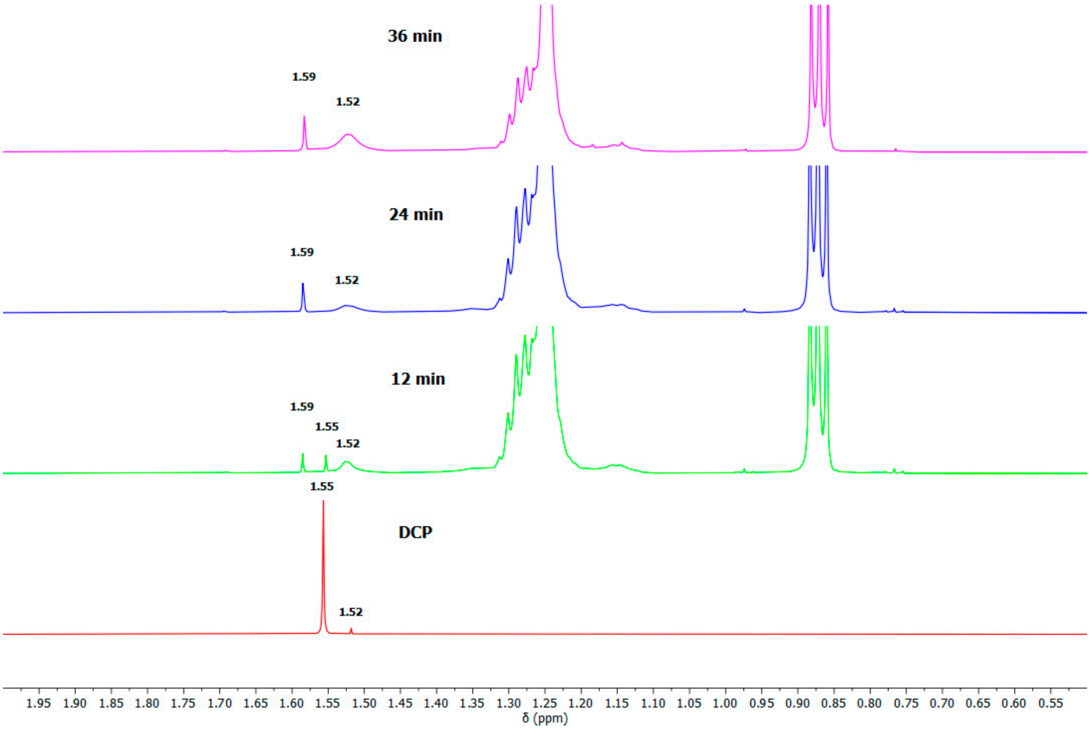


**Figure S4.** $^1$H NMR spectrum of the DCP at RT and $^1$H NMR spectra of the mixture of DCP and DCPD-*bis*TEMPO after 12, 24, and 36 min of heating at 150 °C (Range from 0.5 to 2.0 ppm).

**Table S1.** Mechanical properties of dynamically and conventionally crosslinked LDPE samples processed at 150 °C as a function of the crosslinker concentration.

| Sample designation | Conc. of crosslinker mol % | Young's Modulus GPa | Tensile Strength MPa | Elongation at break % |
|---|---|---|---|---|
| LDPE | 0 | 0,205 ± 0,04 | 17,5 ± 0,6 | 39 ± 3,8 |
| DCPD | 1,45 | 0,205 ± 0,03 | 17,5 ± 0,8 | 43 ± 7,3 |
| DCPD | 2,9 | 0,195 ± 0,03 | 17,4 ± 0,6 | 40 ± 4,5 |
| DCPD | 5,8 | 0,196 ± 0,02 | 17,3 ± 0,6 | 44 ± 8,9 |
| ADK | 1,45 | 0,199 ± 0,02 | 17,8 ± 0,4 | 42 ± 5,2 |
| ADK | 2,9 | 0,206 ± 0,05 | 17,6 ± 0,4 | 39 ± 4,9 |
| ADK | 5,8 | 0,207 ± 0,03 | 17,6 ± 1,1 | 40 ± 5,1 |

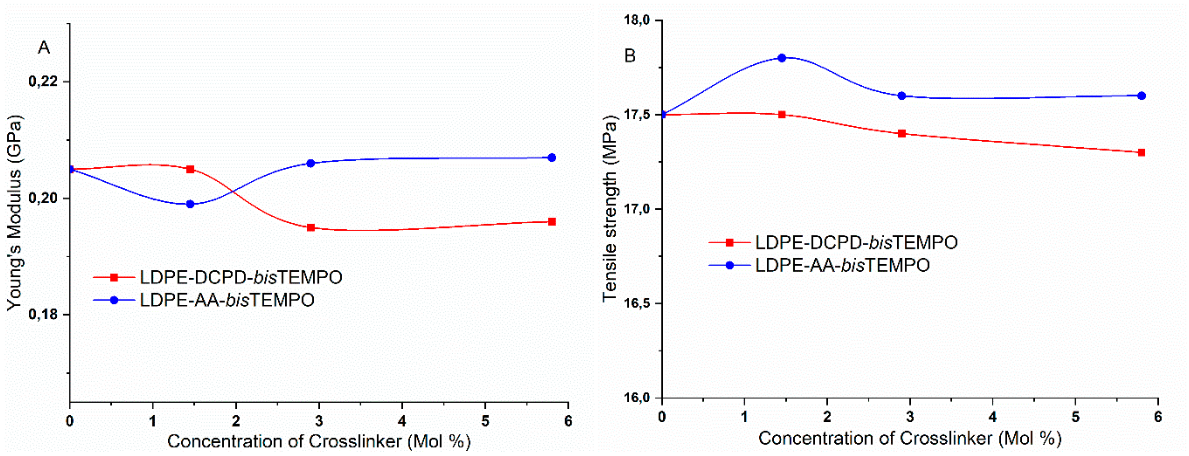


**Figure S5.** Young's modulus (A) and tensile strength (B) of LDPEs processed at 150 °C as a function of the crosslinker concentration (DCPD-*bis*TEMPO or AA-*bis*TEMPO).

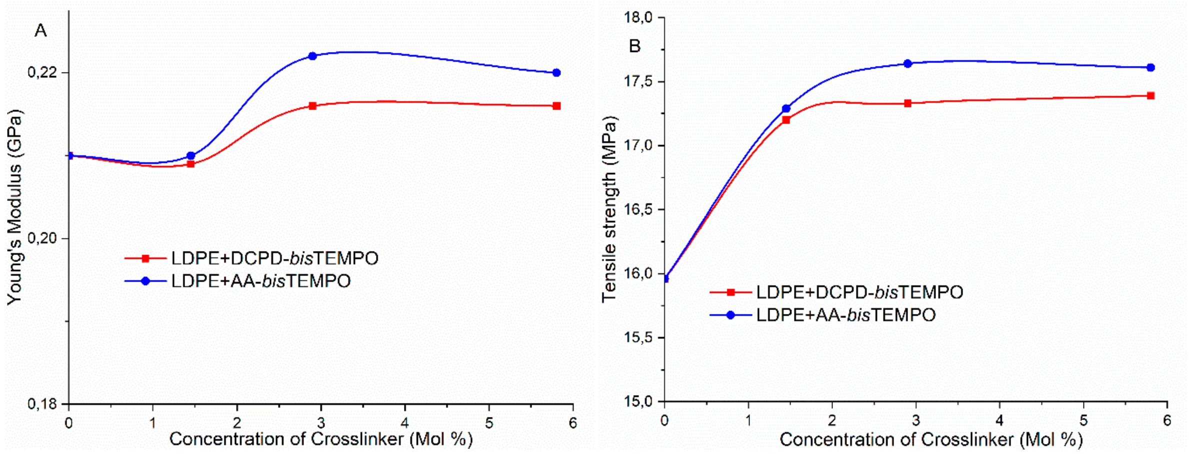


**Figure S6.** Young's modulus (A) and tensile strength (B) of LDPEs processed at 170 °C as a function of the crosslinker concentration (DCPD-*bis*TEMPO or AA-*bis*TEMPO).

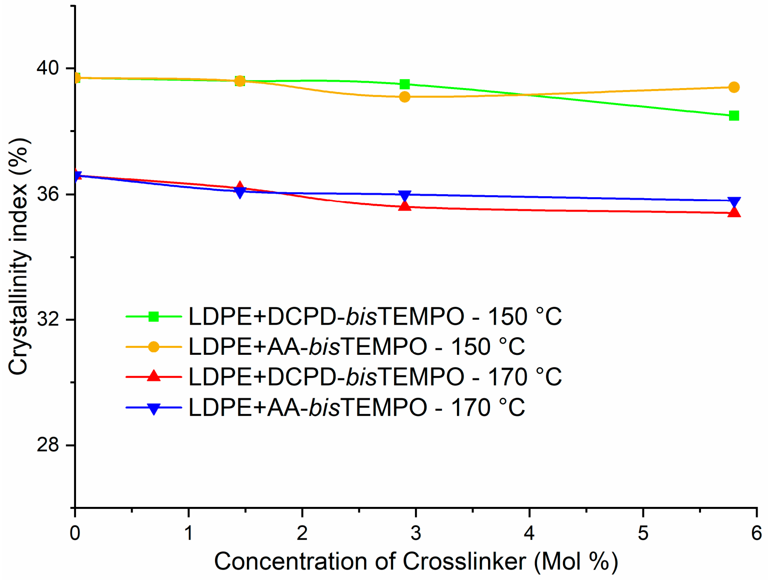


**Figure S7.** Crystallinity index as a function of the crosslinker concentration and the processing temperature.
